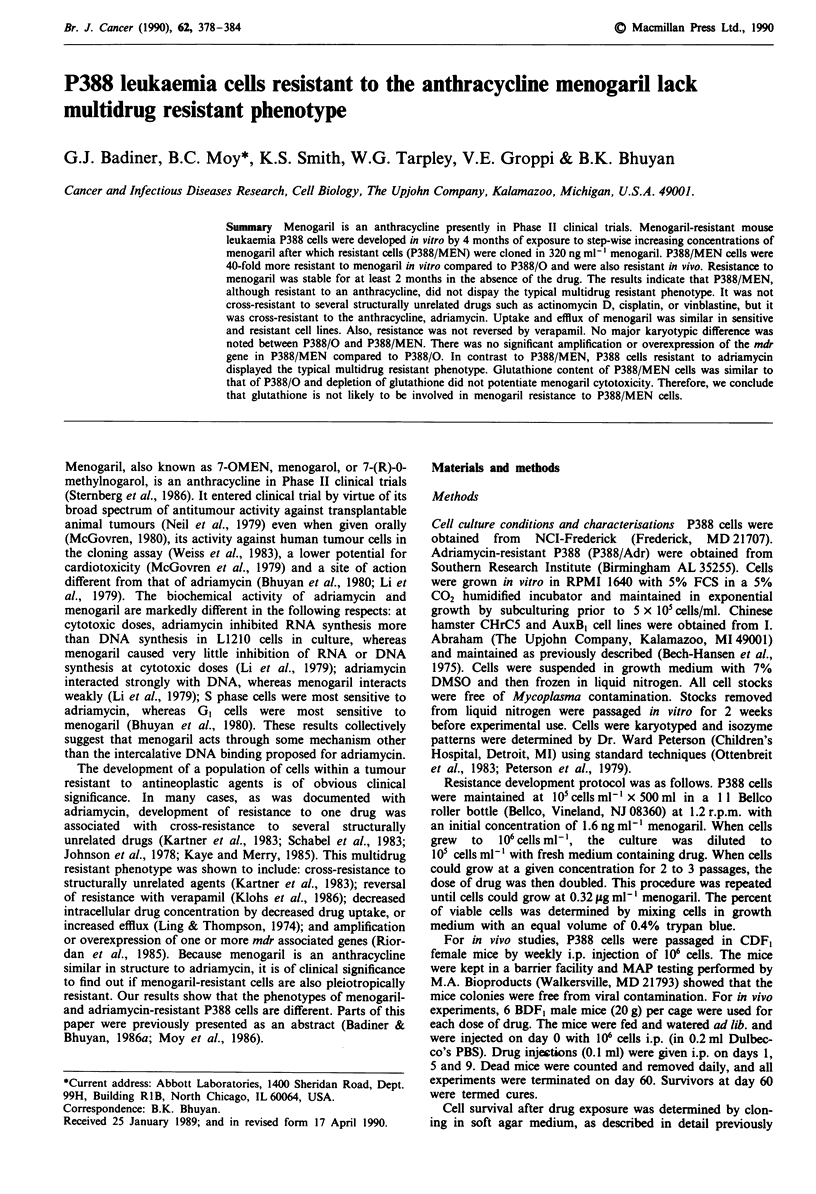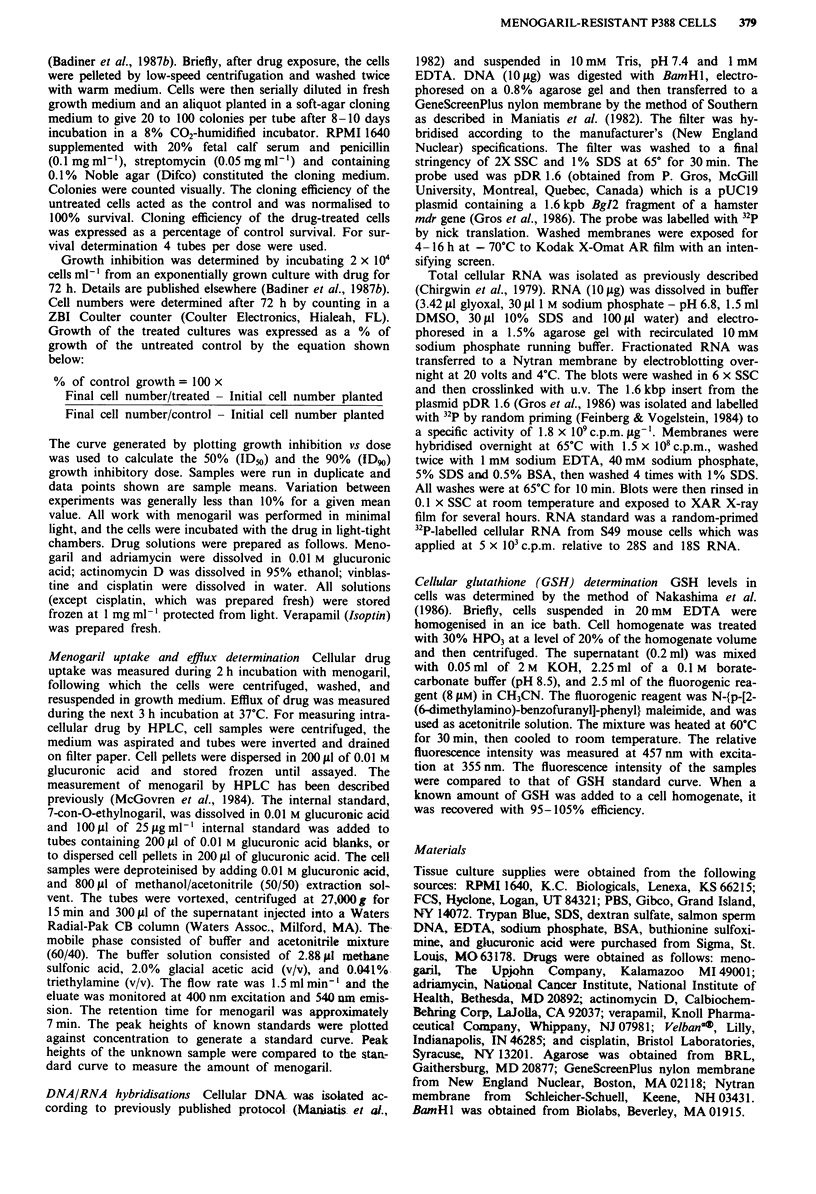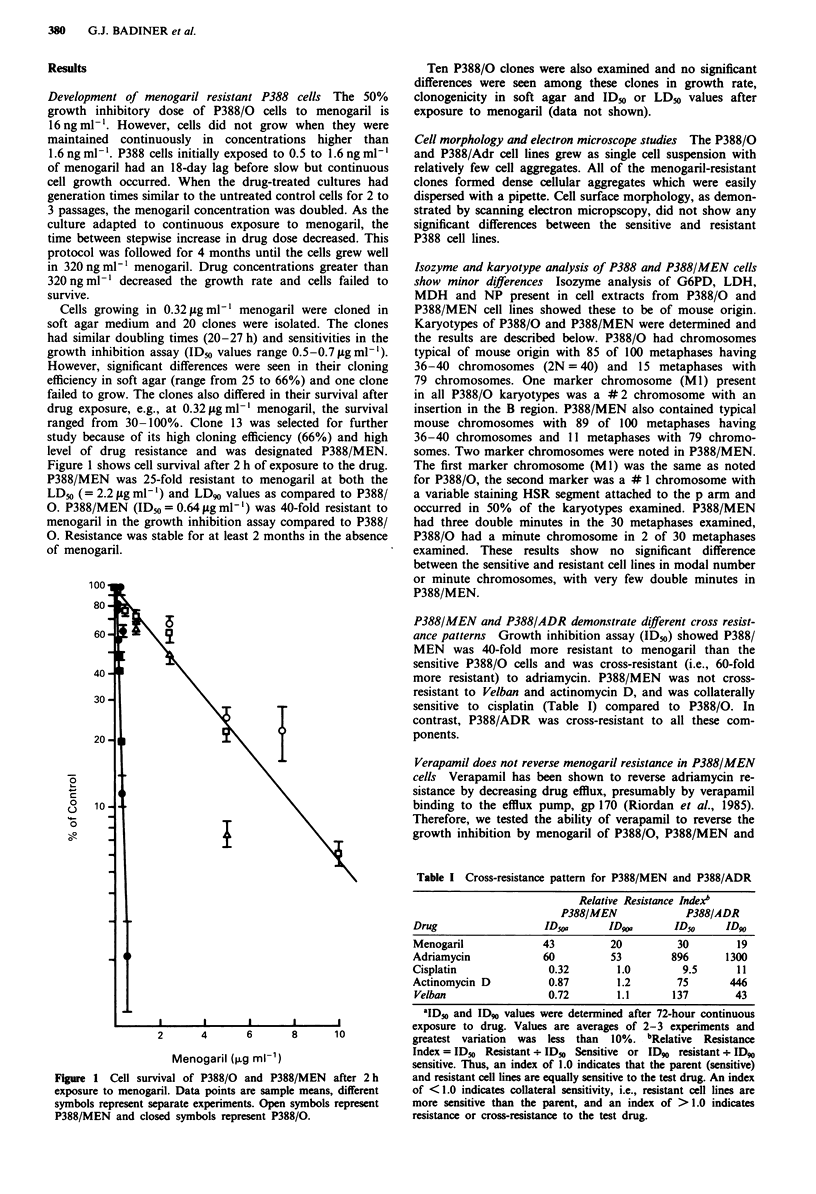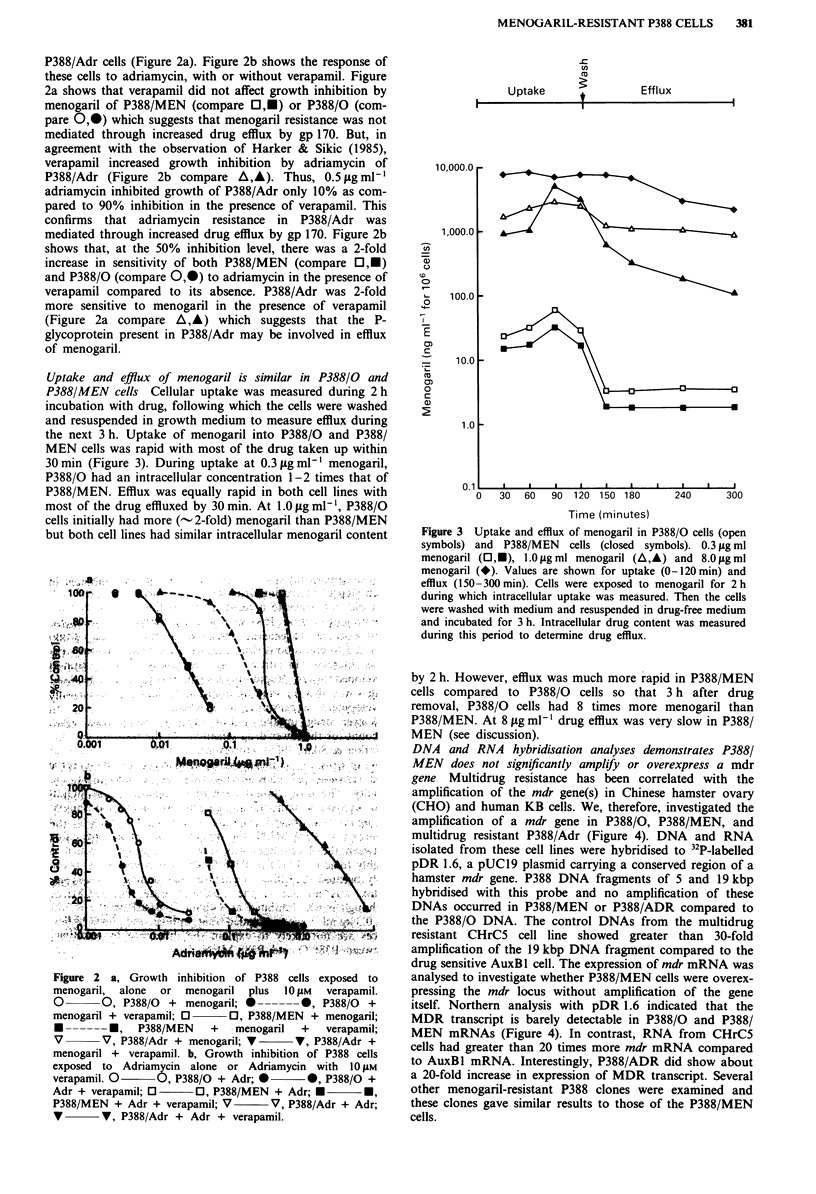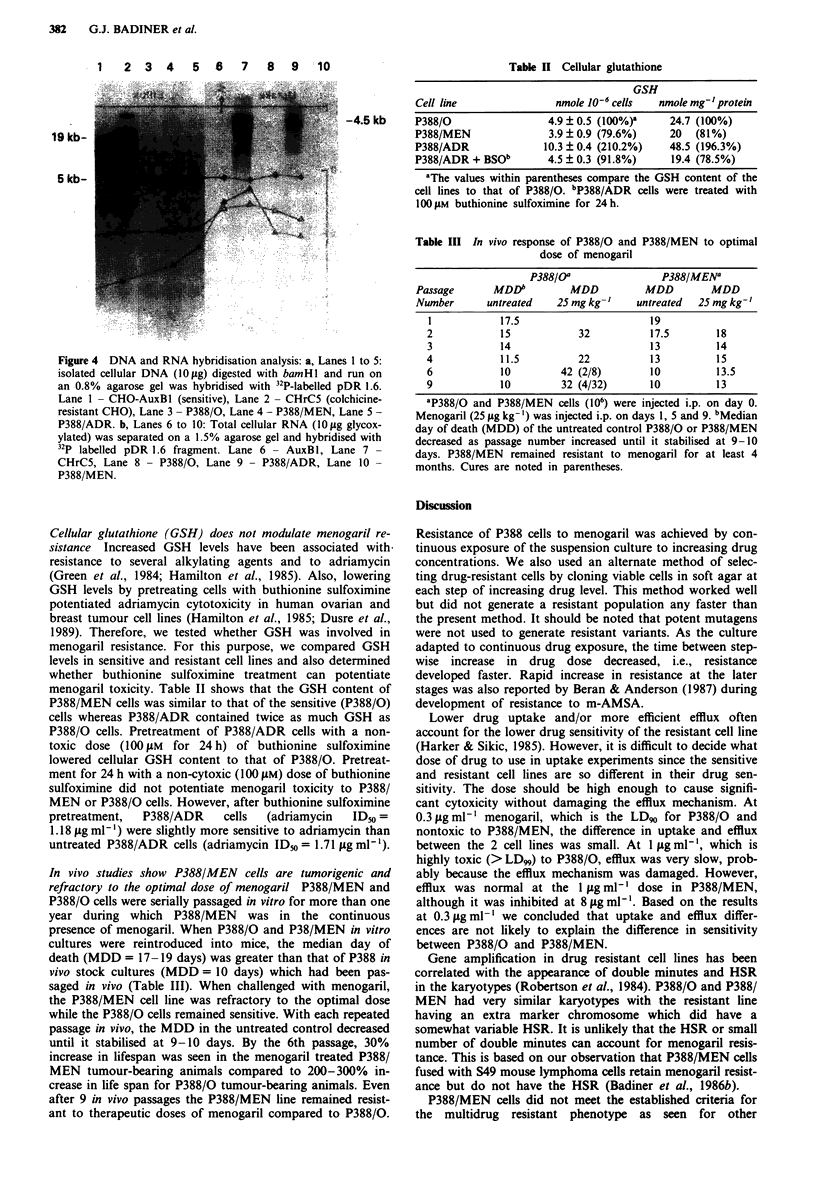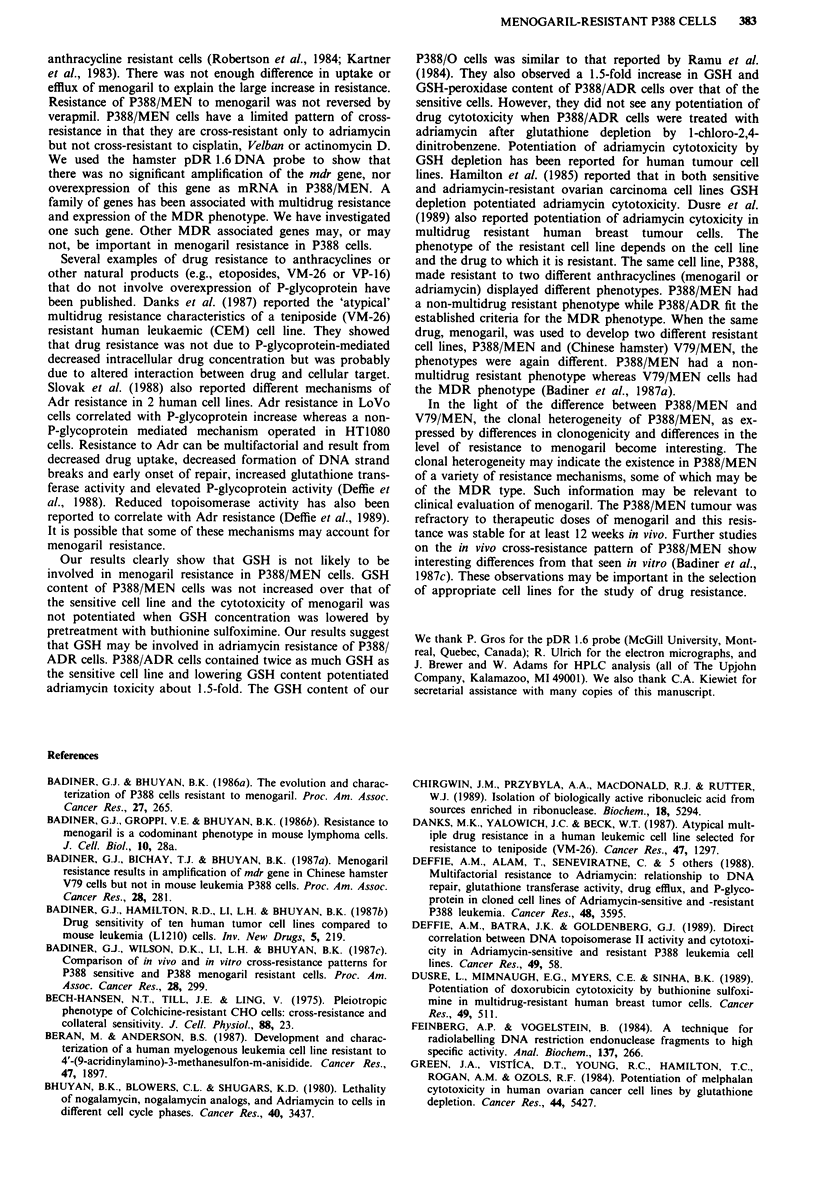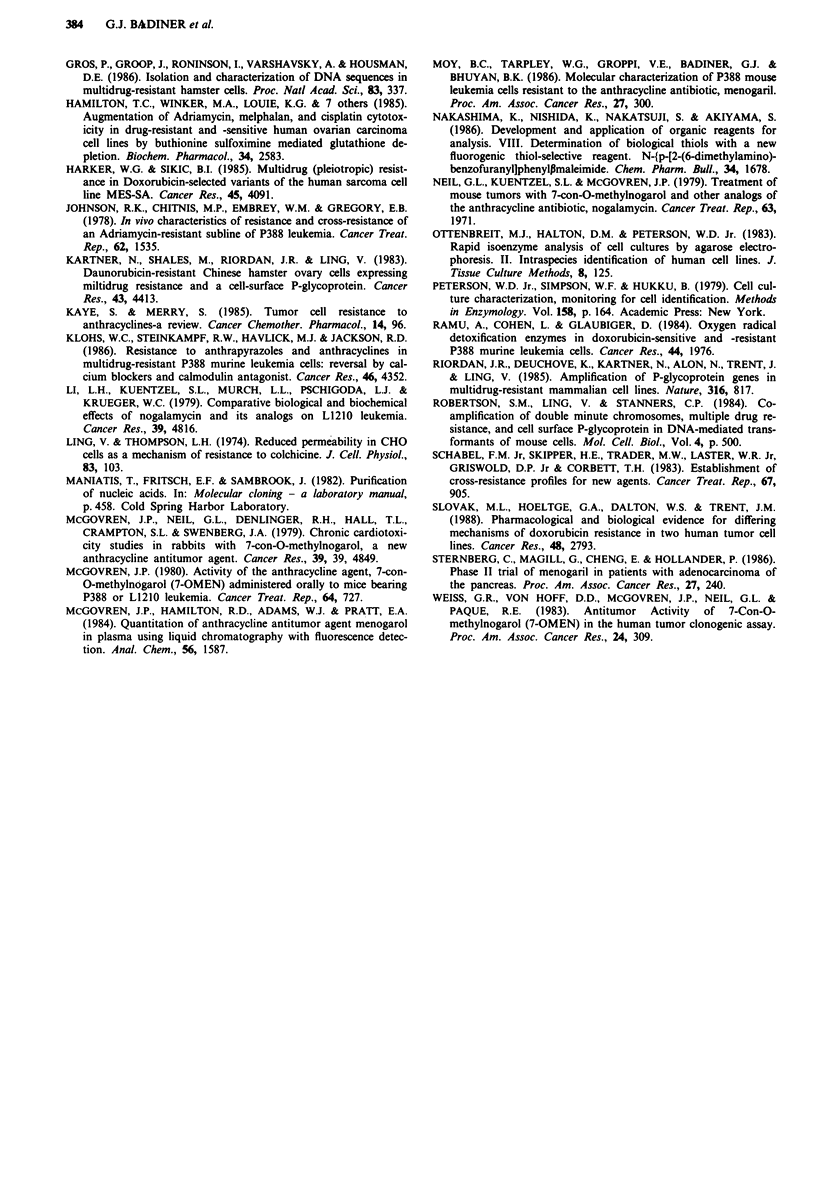# P388 leukaemia cells resistant to the anthracycline menogaril lack multidrug resistant phenotype.

**DOI:** 10.1038/bjc.1990.302

**Published:** 1990-09

**Authors:** G. J. Badiner, B. C. Moy, K. S. Smith, W. G. Tarpley, V. E. Groppi, B. K. Bhuyan

**Affiliations:** Cancer and Infectious Diseases Research, Upjohn Company, Kalamazoo, Michigan 49001.

## Abstract

**Images:**


Br. J. Cancer (1990), 62, 378-384 © Macmillan Press Ltd., 1990

### P388 leukaemia cells resistant to the anthracycline menogaril lack multidrug resistant phenotype

G.J. Badiner, B.C. Moy*, K.S. Smith, W.G. Tarpley, V.E. Groppi & B.K. Bhuyan

*Cancer and Infectious Diseases Research, Cell Biology, The Upjohn Company, Kalamazoo, Michigan, U.S.A. 49001.*

**Summary** Menogaril is an anthracycline presently in Phase II clinical trials. Menogaril-resistant mouse leukaemia P388 cells were developed *in vitro* by 4 months of exposure to step-wise increasing concentrations of menogaril after which resistant cells (P388/MEN) were cloned in 320 ng $ml^{-1}$ menogaril. P388/MEN cells were 40-fold more resistant to menogaril *in vitro* compared to P388/O and were also resistant *in vivo*. Resistance to menogaril was stable for at least 2 months in the absence of the drug. The results indicate that P388/MEN, although resistant to an anthracycline, did not dispay the typical multidrug resistant phenotype. It was not cross-resistant to several structurally unrelated drugs such as actinomycin D, cisplatin, or vinblastine, but it was cross-resistant to the anthracycline, adriamycin. Uptake and efflux of menogaril was similar in sensitive and resistant cell lines. Also, resistance was not reversed by verapamil. No major karyotypic difference was noted between P388/O and P388/MEN. There was no significant amplification or overexpression of the *mdr* gene in P388/MEN compared to P388/O. In contrast to P388/MEN, P388 cells resistant to adriamycin displayed the typical multidrug resistant phenotype. Glutathione content of P388/MEN cells was similar to that of P388/O and depletion of glutathione did not potentiate menogaril cytotoxicity. Therefore, we conclude that glutathione is not likely to be involved in menogaril resistance to P388/MEN cells.

Menogaril, also known as 7-OMEN, menogarol, or 7-(R)-0-methylnogarol, is an anthracycline in Phase II clinical trials (Sternberg *et al.*, 1986). It entered clinical trial by virtue of its broad spectrum of antitumour activity against transplantable animal tumours (Neil *et al.*, 1979) even when given orally (McGovren, 1980), its activity against human tumour cells in the cloning assay (Weiss *et al.*, 1983), a lower potential for cardiotoxicity (McGovren *et al.*, 1979) and a site of action different from that of adriamycin (Bhuyan *et al.*, 1980; Li *et al.*, 1979). The biochemical activity of adriamycin and menogaril are markedly different in the following respects: at cytotoxic doses, adriamycin inhibited RNA synthesis more than DNA synthesis in L1210 cells in culture, whereas menogaril caused very little inhibition of RNA or DNA synthesis at cytotoxic doses (Li *et al.*, 1979); adriamycin interacted strongly with DNA, whereas menogaril interacts weakly (Li *et al.*, 1979); S phase cells were most sensitive to adriamycin, whereas $G_1$ cells were most sensitive to menogaril (Bhuyan *et al.*, 1980). These results collectively suggest that menogaril acts through some mechanism other than the intercalative DNA binding proposed for adriamycin.

The development of a population of cells within a tumour resistant to antineoplastic agents is of obvious clinical significance. In many cases, as was documented with adriamycin, development of resistance to one drug was associated with cross-resistance to several structurally unrelated drugs (Kartner *et al.*, 1983; Schabel *et al.*, 1983; Johnson *et al.*, 1978; Kaye and Merry, 1985). This multidrug resistant phenotype was shown to include: cross-resistance to structurally unrelated agents (Kartner *et al.*, 1983); reversal of resistance with verapamil (Klohs *et al.*, 1986); decreased intracellular drug concentration by decreased drug uptake, or increased efflux (Ling & Thompson, 1974); and amplification or overexpression of one or more *mdr* associated genes (Riordan *et al.*, 1985). Because menogaril is an anthracycline similar in structure to adriamycin, it is of clinical significance to find out if menogaril-resistant cells are also pleiotropically resistant. Our results show that the phenotypes of menogaril- and adriamycin-resistant P388 cells are different. Parts of this paper were previously presented as an abstract (Badiner & Bhuyan, 1986*a*; Moy *et al.*, 1986).

*Current address: Abbott Laboratories, 1400 Sheridan Road, Dept. 99H, Building R1B, North Chicago, IL 60064, USA.
Correspondence: B.K. Bhuyan.
Received 25 January 1989; and in revised form 17 April 1990.

### Materials and methods

### *Methods*

*Cell culture conditions and characterisations* P388 cells were obtained from NCI-Frederick (Frederick, MD 21707). Adriamycin-resistant P388 (P388/Adr) were obtained from Southern Research Institute (Birmingham AL 35255). Cells were grown *in vitro* in RPMI 1640 with 5% FCS in a 5% $CO_2$ humidified incubator and maintained in exponential growth by subculturing prior to $5 \times 10^5$ cells/ml. Chinese hamster CHrC5 and $AuxB_1$ cell lines were obtained from I. Abraham (The Upjohn Company, Kalamazoo, MI 49001) and maintained as previously described (Bech-Hansen *et al.*, 1975). Cells were suspended in growth medium with 7% DMSO and then frozen in liquid nitrogen. All cell stocks were free of *Mycoplasma* contamination. Stocks removed from liquid nitrogen were passaged *in vitro* for 2 weeks before experimental use. Cells were karyotyped and isozyme patterns were determined by Dr. Ward Peterson (Children's Hospital, Detroit, MI) using standard techniques (Ottenbreit *et al.*, 1983; Peterson *et al.*, 1979).

Resistance development protocol was as follows. P388 cells were maintained at $10^5$ cells $ml^{-1} \times 500$ ml in a 1 l Bellco roller bottle (Bellco, Vineland, NJ 08360) at 1.2 r.p.m. with an initial concentration of 1.6 ng $ml^{-1}$ menogaril. When cells grew to $10^6$ cells $ml^{-1}$, the culture was diluted to $10^5$ cells $ml^{-1}$ with fresh medium containing drug. When cells could grow at a given concentration for 2 to 3 passages, the dose of drug was then doubled. This procedure was repeated until cells could grow at 0.32 $\mu$g $ml^{-1}$ menogaril. The percent of viable cells was determined by mixing cells in growth medium with an equal volume of 0.4% trypan blue.

For *in vivo* studies, P388 cells were passaged in $CDF_1$ female mice by weekly i.p. injection of $10^6$ cells. The mice were kept in a barrier facility and MAP testing performed by M.A. Bioproducts (Walkersville, MD 21793) showed that the mice colonies were free from viral contamination. For *in vivo* experiments, 6 $BDF_1$ male mice (20 g) per cage were used for each dose of drug. The mice were fed and watered *ad lib.* and were injected on day 0 with $10^6$ cells i.p. (in 0.2 ml Dulbecco's PBS). Drug injections (0.1 ml) were given i.p. on days 1, 5 and 9. Dead mice were counted and removed daily, and all experiments were terminated on day 60. Survivors at day 60 were termed cures.

Cell survival after drug exposure was determined by cloning in soft agar medium, as described in detail previously

(Badiner *et al.*, 1987*b*). Briefly, after drug exposure, the cells were pelleted by low-speed centrifugation and washed twice with warm medium. Cells were then serially diluted in fresh growth medium and an aliquot planted in a soft-agar cloning medium to give 20 to 100 colonies per tube after 8–10 days incubation in a 8% $CO_2$-humidified incubator. RPMI 1640 supplemented with 20% fetal calf serum and penicillin (0.1 mg ml$^{-1}$), streptomycin (0.05 mg ml$^{-1}$) and containing 0.1% Noble agar (Difco) constituted the cloning medium. Colonies were counted visually. The cloning efficiency of the untreated cells acted as the control and was normalised to 100% survival. Cloning efficiency of the drug-treated cells was expressed as a percentage of control survival. For survival determination 4 tubes per dose were used.

Growth inhibition was determined by incubating $2 \times 10^4$ cells ml$^{-1}$ from an exponentially grown culture with drug for 72 h. Details are published elsewhere (Badiner *et al.*, 1987*b*). Cell numbers were determined after 72 h by counting in a ZBI Coulter counter (Coulter Electronics, Hialeah, FL). Growth of the treated cultures was expressed as a % of growth of the untreated control by the equation shown below:

$$\% \text{ of control growth} = 100 \times \frac{\text{Final cell number/treated} - \text{Initial cell number planted}}{\text{Final cell number/control} - \text{Initial cell number planted}}$$

The curve generated by plotting growth inhibition *vs* dose was used to calculate the 50% ($ID_{50}$) and the 90% ($ID_{90}$) growth inhibitory dose. Samples were run in duplicate and data points shown are sample means. Variation between experiments was generally less than 10% for a given mean value. All work with menogaril was performed in minimal light, and the cells were incubated with the drug in light-tight chambers. Drug solutions were prepared as follows. Menogaril and adriamycin were dissolved in 0.01 M glucuronic acid; actinomycin D was dissolved in 95% ethanol; vinblastine and cisplatin were dissolved in water. All solutions (except cisplatin, which was prepared fresh) were stored frozen at 1 mg ml$^{-1}$ protected from light. Verapamil (*Isoptin*) was prepared fresh.

*Menogaril uptake and efflux determination* Cellular drug uptake was measured during 2 h incubation with menogaril, following which the cells were centrifuged, washed, and resuspended in growth medium. Efflux of drug was measured during the next 3 h incubation at 37°C. For measuring intracellular drug by HPLC, cell samples were centrifuged, the medium was aspirated and tubes were inverted and drained on filter paper. Cell pellets were dispersed in 200 µl of 0.01 M glucuronic acid and stored frozen until assayed. The measurement of menogaril by HPLC has been described previously (McGovren *et al.*, 1984). The internal standard, 7-con-O-ethylnogaril, was dissolved in 0.01 M glucuronic acid and 100 µl of 25 µg ml$^{-1}$ internal standard was added to tubes containing 200 µl of 0.01 M glucuronic acid blanks, or to dispersed cell pellets in 200 µl of glucuronic acid. The cell samples were deproteinised by adding 0.01 M glucuronic acid, and 800 µl of methanol/acetonitrile (50/50) extraction solvent. The tubes were vortexed, centrifuged at 27,000 *g* for 15 min and 300 µl of the supernatant injected into a Waters Radial-Pak CB column (Waters Assoc., Milford, MA). The mobile phase consisted of buffer and acetonitrile mixture (60/40). The buffer solution consisted of 2.88 µl methane sulfonic acid, 2.0% glacial acetic acid (v/v), and 0.041% triethylamine (v/v). The flow rate was 1.5 ml min$^{-1}$ and the eluate was monitored at 400 nm excitation and 540 nm emission. The retention time for menogaril was approximately 7 min. The peak heights of known standards were plotted against concentration to generate a standard curve. Peak heights of the unknown sample were compared to the standard curve to measure the amount of menogaril.

*DNA/RNA hybridisations* Cellular DNA was isolated according to previously published protocol (Maniatis *et al.*, 1982) and suspended in 10 mM Tris, pH 7.4 and 1 mM EDTA. DNA (10 µg) was digested with *Bam*H1, electrophoresed on a 0.8% agarose gel and then transferred to a GeneScreenPlus nylon membrane by the method of Southern as described in Maniatis *et al.* (1982). The filter was hybridised according to the manufacturer's (New England Nuclear) specifications. The filter was washed to a final stringency of 2X SSC and 1% SDS at 65° for 30 min. The probe used was pDR 1.6 (obtained from P. Gros, McGill University, Montreal, Quebec, Canada) which is a pUC19 plasmid containing a 1.6 kpb *Bgl2* fragment of a hamster *mdr* gene (Gros *et al.*, 1986). The probe was labelled with $^{32}P$ by nick translation. Washed membranes were exposed for 4–16 h at − 70°C to Kodak X-Omat AR film with an intensifying screen.

Total cellular RNA was isolated as previously described (Chirgwin *et al.*, 1979). RNA (10 µg) was dissolved in buffer (3.42 µl glyoxal, 30 µl 1 M sodium phosphate – pH 6.8, 1.5 ml DMSO, 30 µl 10% SDS and 100 µl water) and electrophoresed in a 1.5% agarose gel with recirculated 10 mM sodium phosphate running buffer. Fractionated RNA was transferred to a Nytran membrane by electroblotting overnight at 20 volts and 4°C. The blots were washed in 6 × SSC and then crosslinked with u.v. The 1.6 kbp insert from the plasmid pDR 1.6 (Gros *et al.*, 1986) was isolated and labelled with $^{32}P$ by random priming (Feinberg & Vogelstein, 1984) to a specific activity of $1.8 \times 10^9$ c.p.m. µg$^{-1}$. Membranes were hybridised overnight at 65°C with $1.5 \times 10^8$ c.p.m., washed twice with 1 mM sodium EDTA, 40 mM sodium phosphate, 5% SDS and 0.5% BSA, then washed 4 times with 1% SDS. All washes were at 65°C for 10 min. Blots were then rinsed in 0.1 × SSC at room temperature and exposed to XAR X-ray film for several hours. RNA standard was a random-primed $^{32}P$-labelled cellular RNA from S49 mouse cells which was applied at $5 \times 10^3$ c.p.m. relative to 28S and 18S RNA.

*Cellular glutathione (GSH) determination* GSH levels in cells was determined by the method of Nakashima *et al.* (1986). Briefly, cells suspended in 20 mM EDTA were homogenised in an ice bath. Cell homogenate was treated with 30% $HPO_3$ at a level of 20% of the homogenate volume and then centrifuged. The supernatant (0.2 ml) was mixed with 0.05 ml of 2 M KOH, 2.25 ml of a 0.1 M borate-carbonate buffer (pH 8.5), and 2.5 ml of the fluorogenic reagent (8 µM) in $CH_3CN$. The fluorogenic reagent was N-{p-[2-(6-dimethylamino)-benzofuranyl]-phenyl} maleimide, and was used as acetonitrile solution. The mixture was heated at 60°C for 30 min, then cooled to room temperature. The relative fluorescence intensity was measured at 457 nm with excitation at 355 nm. The fluorescence intensity of the samples were compared to that of GSH standard curve. When a known amount of GSH was added to a cell homogenate, it was recovered with 95–105% efficiency.

### *Materials*

Tissue culture supplies were obtained from the following sources: RPMI 1640, K.C. Biologicals, Lenexa, KS 66215; FCS, Hyclone, Logan, UT 84321; PBS, Gibco, Grand Island, NY 14072. Trypan Blue, SDS, dextran sulfate, salmon sperm DNA, EDTA, sodium phosphate, BSA, buthionine sulfoximine, and glucuronic acid were purchased from Sigma, St. Louis, MO 63178. Drugs were obtained as follows: menogaril, The Upjohn Company, Kalamazoo MI 49001; adriamycin, National Cancer Institute, National Institute of Health, Bethesda, MD 20892; actinomycin D, Calbiochem-Behring Corp, LaJolla, CA 92037; verapamil, Knoll Pharmaceutical Company, Whippany, NJ 07981; *Velban*®, Lilly, Indianapolis, IN 46285; and cisplatin, Bristol Laboratories, Syracuse, NY 13201. Agarose was obtained from BRL, Gaithersburg, MD 20877; GeneScreenPlus nylon membrane from New England Nuclear, Boston, MA 02118; Nytran membrane from Schleicher-Schuell, Keene, NH 03431. *Bam*H1 was obtained from Biolabs, Beverley, MA 01915.

### Results

*Development of menogaril resistant P388 cells* The 50% growth inhibitory dose of P388/O cells to menogaril is 16 ng ml$^{-1}$. However, cells did not grow when they were maintained continuously in concentrations higher than 1.6 ng ml$^{-1}$. P388 cells initially exposed to 0.5 to 1.6 ng ml$^{-1}$ of menogaril had an 18-day lag before slow but continuous cell growth occurred. When the drug-treated cultures had generation times similar to the untreated control cells for 2 to 3 passages, the menogaril concentration was doubled. As the culture adapted to continuous exposure to menogaril, the time between stepwise increase in drug dose decreased. This protocol was followed for 4 months until the cells grew well in 320 ng ml$^{-1}$ menogaril. Drug concentrations greater than 320 ng ml$^{-1}$ decreased the growth rate and cells failed to survive.

Cells growing in 0.32 μg ml$^{-1}$ menogaril were cloned in soft agar medium and 20 clones were isolated. The clones had similar doubling times (20–27 h) and sensitivities in the growth inhibition assay ($ID_{50}$ values range 0.5–0.7 μg ml$^{-1}$). However, significant differences were seen in their cloning efficiency in soft agar (range from 25 to 66%) and one clone failed to grow. The clones also differed in their survival after drug exposure, e.g., at 0.32 μg ml$^{-1}$ menogaril, the survival ranged from 30–100%. Clone 13 was selected for further study because of its high cloning efficiency (66%) and high level of drug resistance and was designated P388/MEN. Figure 1 shows cell survival after 2 h of exposure to the drug. P388/MEN was 25-fold resistant to menogaril at both the $LD_{50}$ (= 2.2 μg ml$^{-1}$) and $LD_{90}$ values as compared to P388/O. P388/MEN ($ID_{50}$ = 0.64 μg ml$^{-1}$) was 40-fold resistant to menogaril in the growth inhibition assay compared to P388/O. Resistance was stable for at least 2 months in the absence of menogaril.

**Figure 1** Cell survival of P388/O and P388/MEN after 2 h exposure to menogaril. Data points are sample means, different symbols represent separate experiments. Open symbols represent P388/MEN and closed symbols represent P388/O.

Ten P388/O clones were also examined and no significant differences were seen among these clones in growth rate, clonogenicity in soft agar and $ID_{50}$ or $LD_{50}$ values after exposure to menogaril (data not shown).

*Cell morphology and electron microscope studies* The P388/O and P388/Adr cell lines grew as single cell suspension with relatively few cell aggregates. All of the menogaril-resistant clones formed dense cellular aggregates which were easily dispersed with a pipette. Cell surface morphology, as demonstrated by scanning electron micropscopy, did not show any significant differences between the sensitive and resistant P388 cell lines.

*Isozyme and karyotype analysis of P388 and P388/MEN cells show minor differences* Isozyme analysis of G6PD, LDH, MDH and NP present in cell extracts from P388/O and P388/MEN cell lines showed these to be of mouse origin. Karyotypes of P388/O and P388/MEN were determined and the results are described below. P388/O had chromosomes typical of mouse origin with 85 of 100 metaphases having 36–40 chromosomes (2N = 40) and 15 metaphases with 79 chromosomes. One marker chromosome (M1) present in all P388/O karyotypes was a #2 chromosome with an insertion in the B region. P388/MEN also contained typical mouse chromosomes with 89 of 100 metaphases having 36–40 chromosomes and 11 metaphases with 79 chromosomes. Two marker chromosomes were noted in P388/MEN. The first marker chromosome (M1) was the same as noted for P388/O, the second marker was a #1 chromosome with a variable staining HSR segment attached to the p arm and occurred in 50% of the karyotypes examined. P388/MEN had three double minutes in the 30 metaphases examined, P388/O had a minute chromosome in 2 of 30 metaphases examined. These results show no significant difference between the sensitive and resistant cell lines in modal number or minute chromosomes, with very few double minutes in P388/MEN.

*P388/MEN and P388/ADR demonstrate different cross resistance patterns* Growth inhibition assay ($ID_{50}$) showed P388/MEN was 40-fold more resistant to menogaril than the sensitive P388/O cells and was cross-resistant (i.e., 60-fold more resistant) to adriamycin. P388/MEN was not cross-resistant to *Velban* and actinomycin D, and was collaterally sensitive to cisplatin (Table I) compared to P388/O. In contrast, P388/ADR was cross-resistant to all these components.

*Verapamil does not reverse menogaril resistance in P388/MEN cells* Verapamil has been shown to reverse adriamycin resistance by decreasing drug efflux, presumably by verapamil binding to the efflux pump, gp 170 (Riordan *et al.*, 1985). Therefore, we tested the ability of verapamil to reverse the growth inhibition by menogaril of P388/O, P388/MEN and

**Table I** Cross-resistance pattern for P388/MEN and P388/ADR

| | *Relative Resistance Index*[b] | | | |
|---|---|---|---|---|
| | *P388/MEN* | | *P388/ADR* | |
| *Drug* | $ID_{50}$[a] | $ID_{90}$[a] | $ID_{50}$ | $ID_{90}$ |
| Menogaril | 43 | 20 | 30 | 19 |
| Adriamycin | 60 | 53 | 896 | 1300 |
| Cisplatin | 0.32 | 1.0 | 9.5 | 11 |
| Actinomycin D | 0.87 | 1.2 | 75 | 446 |
| *Velban* | 0.72 | 1.1 | 137 | 43 |

[a]$ID_{50}$ and $ID_{90}$ values were determined after 72-hour continuous exposure to drug. Values are averages of 2–3 experiments and greatest variation was less than 10%. [b]Relative Resistance Index = $ID_{50}$ Resistant ÷ $ID_{50}$ Sensitive or $ID_{90}$ resistant ÷ $ID_{90}$ sensitive. Thus, an index of 1.0 indicates that the parent (sensitive) and resistant cell lines are equally sensitive to the test drug. An index of < 1.0 indicates collateral sensitivity, i.e., resistant cell lines are more sensitive than the parent, and an index of > 1.0 indicates resistance or cross-resistance to the test drug.

P388/Adr cells (Figure 2a). Figure 2b shows the response of these cells to adriamycin, with or without verapamil. Figure 2a shows that verapamil did not affect growth inhibition by menogaril of P388/MEN (compare □,■) or P388/O (compare ○,●) which suggests that menogaril resistance was not mediated through increased drug efflux by gp 170. But, in agreement with the observation of Harker & Sikic (1985), verapamil increased growth inhibition by adriamycin of P388/Adr (Figure 2b compare Δ,▲). Thus, 0.5 μg ml$^{-1}$ adriamycin inhibited growth of P388/Adr only 10% as compared to 90% inhibition in the presence of verapamil. This confirms that adriamycin resistance in P388/Adr was mediated through increased drug efflux by gp 170. Figure 2b shows that, at the 50% inhibition level, there was a 2-fold increase in sensitivity of both P388/MEN (compare □,■) and P388/O (compare ○,●) to adriamycin in the presence of verapamil compared to its absence. P388/Adr was 2-fold more sensitive to menogaril in the presence of verapamil (Figure 2a compare Δ,▲) which suggests that the P-glycoprotein present in P388/Adr may be involved in efflux of menogaril.

*Uptake and efflux of menogaril is similar in P388/O and P388/MEN cells* Cellular uptake was measured during 2 h incubation with drug, following which the cells were washed and resuspended in growth medium to measure efflux during the next 3 h. Uptake of menogaril into P388/O and P388/MEN cells was rapid with most of the drug taken up within 30 min (Figure 3). During uptake at 0.3 μg ml$^{-1}$ menogaril, P388/O had an intracellular concentration 1–2 times that of P388/MEN. Efflux was equally rapid in both cell lines with most of the drug effluxed by 30 min. At 1.0 μg ml$^{-1}$, P388/O cells initially had more (~2-fold) menogaril than P388/MEN but both cell lines had similar intracellular menogaril content

**Figure 2** **a**, Growth inhibition of P388 cells exposed to menogaril, alone or menogaril plus 10 μM verapamil. ○——○, P388/O + menogaril; ●------●, P388/O + menogaril + verapamil; □——□, P388/MEN + menogaril; ■------■, P388/MEN + menogaril + verapamil; ∇——∇, P388/Adr + menogaril; ▼——▼, P388/Adr + menogaril + verapamil. **b**, Growth inhibition of P388 cells exposed to Adriamycin alone or Adriamycin with 10 μM verapamil. ○——○, P388/O + Adr; ●——●, P388/O + Adr + verapamil; □——□, P388/MEN + Adr; ■——■, P388/MEN + Adr + verapamil; ∇——∇, P388/Adr + Adr; ▼——▼, P388/Adr + Adr + verapamil.

**Figure 3** Uptake and efflux of menogaril in P388/O cells (open symbols) and P388/MEN cells (closed symbols). 0.3 μg ml menogaril (□,■), 1.0 μg ml menogaril (Δ,▲) and 8.0 μg ml menogaril (◆). Values are shown for uptake (0–120 min) and efflux (150–300 min). Cells were exposed to menogaril for 2 h during which intracellular uptake was measured. Then the cells were washed with medium and resuspended in drug-free medium and incubated for 3 h. Intracellular drug content was measured during this period to determine drug efflux.

by 2 h. However, efflux was much more rapid in P388/MEN cells compared to P388/O cells so that 3 h after drug removal, P388/O cells had 8 times more menogaril than P388/MEN. At 8 μg ml$^{-1}$ drug efflux was very slow in P388/MEN (see discussion).

*DNA and RNA hybridisation analyses demonstrates P388/MEN does not significantly amplify or overexpress a* mdr *gene* Multidrug resistance has been correlated with the amplification of the *mdr* gene(s) in Chinese hamster ovary (CHO) and human KB cells. We, therefore, investigated the amplification of a *mdr* gene in P388/O, P388/MEN, and multidrug resistant P388/Adr (Figure 4). DNA and RNA isolated from these cell lines were hybridised to $^{32}$P-labelled pDR 1.6, a pUC19 plasmid carrying a conserved region of a hamster *mdr* gene. P388 DNA fragments of 5 and 19 kbp hybridised with this probe and no amplification of these DNAs occurred in P388/MEN or P388/ADR compared to the P388/O DNA. The control DNAs from the multidrug resistant CHrC5 cell line showed greater than 30-fold amplification of the 19 kbp DNA fragment compared to the drug sensitive AuxB1 cell. The expression of *mdr* mRNA was analysed to investigate whether P388/MEN cells were overexpressing the *mdr* locus without amplification of the gene itself. Northern analysis with pDR 1.6 indicated that the MDR transcript is barely detectable in P388/O and P388/MEN mRNAs (Figure 4). In contrast, RNA from CHrC5 cells had greater than 20 times more *mdr* mRNA compared to AuxB1 mRNA. Interestingly, P388/ADR did show about a 20-fold increase in expression of MDR transcript. Several other menogaril-resistant P388 clones were examined and these clones gave similar results to those of the P388/MEN cells.

**Figure 4** DNA and RNA hybridisation analysis: **a**, Lanes 1 to 5: isolated cellular DNA (10 µg) digested with *bam*H1 and run on an 0.8% agarose gel was hybridised with $^{32}$P-labelled pDR 1.6. Lane 1 – CHO-AuxB1 (sensitive), Lane 2 – CHrC5 (colchicine-resistant CHO), Lane 3 – P388/O, Lane 4 – P388/MEN, Lane 5 – P388/ADR. **b**, Lanes 6 to 10: Total cellular RNA (10 µg glycoxylated) was separated on a 1.5% agarose gel and hybridised with $^{32}$P labelled pDR 1.6 fragment. Lane 6 – AuxB1, Lane 7 – CHrC5, Lane 8 – P388/O, Lane 9 – P388/ADR, Lane 10 – P388/MEN.

*Cellular glutathione (GSH) does not modulate menogaril resistance* Increased GSH levels have been associated with resistance to several alkylating agents and to adriamycin (Green *et al.*, 1984; Hamilton *et al.*, 1985). Also, lowering GSH levels by pretreating cells with buthionine sulfoximine potentiated adriamycin cytotoxicity in human ovarian and breast tumour cell lines (Hamilton *et al.*, 1985; Dusre *et al.*, 1989). Therefore, we tested whether GSH was involved in menogaril resistance. For this purpose, we compared GSH levels in sensitive and resistant cell lines and also determined whether buthionine sulfoximine treatment can potentiate menogaril toxicity. Table II shows that the GSH content of P388/MEN cells was similar to that of the sensitive (P388/O) cells whereas P388/ADR contained twice as much GSH as P388/O cells. Pretreatment of P388/ADR cells with a non-toxic dose (100 µM for 24 h) of buthionine sulfoximine lowered cellular GSH content to that of P388/O. Pretreatment for 24 h with a non-cytoxic (100 µM) dose of buthionine sulfoximine did not potentiate menogaril toxicity to P388/MEN or P388/O cells. However, after buthionine sulfoximine pretreatment, P388/ADR cells (adriamycin $ID_{50}$ = 1.18 µg ml$^{-1}$) were slightly more sensitive to adriamycin than untreated P388/ADR cells (adriamycin $ID_{50}$ = 1.71 µg ml$^{-1}$).

*In vivo studies show P388/MEN cells are tumorigenic and refractory to the optimal dose of menogaril* P388/MEN and P388/O cells were serially passaged *in vitro* for more than one year during which P388/MEN was in the continuous presence of menogaril. When P388/O and P38/MEN *in vitro* cultures were reintroduced into mice, the median day of death (MDD = 17–19 days) was greater than that of P388 *in vivo* stock cultures (MDD = 10 days) which had been passaged *in vivo* (Table III). When challenged with menogaril, the P388/MEN cell line was refractory to the optimal dose while the P388/O cells remained sensitive. With each repeated passage *in vivo*, the MDD in the untreated control decreased until it stabilised at 9–10 days. By the 6th passage, 30% increase in lifespan was seen in the menogaril treated P388/MEN tumour-bearing animals compared to 200–300% increase in life span for P388/O tumour-bearing animals. Even after 9 *in vivo* passages the P388/MEN line remained resistant to therapeutic doses of menogaril compared to P388/O.

**Table II** Cellular glutathione

| *Cell line* | *GSH* nmole $10^{-6}$ cells | *GSH* nmole mg$^{-1}$ protein |
|---|---|---|
| P388/O | 4.9 ± 0.5 (100%)[a] | 24.7 (100%) |
| P388/MEN | 3.9 ± 0.9 (79.6%) | 20 (81%) |
| P388/ADR | 10.3 ± 0.4 (210.2%) | 48.5 (196.3%) |
| P388/ADR + BSO[b] | 4.5 ± 0.3 (91.8%) | 19.4 (78.5%) |

[a]The values within parentheses compare the GSH content of the cell lines to that of P388/O. [b]P388/ADR cells were treated with 100 µM buthionine sulfoximine for 24 h.

**Table III** *In vivo* response of P388/O and P388/MEN to optimal dose of menogaril

| *Passage Number* | *P388/O*[a] *MDD*[b] *untreated* | *P388/O*[a] *MDD 25 mg kg*$^{-1}$ | *P388/MEN*[a] *MDD untreated* | *P388/MEN*[a] *MDD 25 mg kg*$^{-1}$ |
|---|---|---|---|---|
| 1 | 17.5 | | 19 | |
| 2 | 15 | 32 | 17.5 | 18 |
| 3 | 14 | | 13 | 14 |
| 4 | 11.5 | 22 | 13 | 15 |
| 6 | 10 | 42 (2/8) | 10 | 13.5 |
| 9 | 10 | 32 (4/32) | 10 | 13 |

[a]P388/O and P388/MEN cells ($10^6$) were injected i.p. on day 0. Menogaril (25 µg kg$^{-1}$) was injected i.p. on days 1, 5 and 9. [b]Median day of death (MDD) of the untreated control P388/O or P388/MEN decreased as passage number increased until it stabilised at 9–10 days. P388/MEN remained resistant to menogaril for at least 4 months. Cures are noted in parentheses.

### Discussion

Resistance of P388 cells to menogaril was achieved by continuous exposure of the suspension culture to increasing drug concentrations. We also used an alternate method of selecting drug-resistant cells by cloning viable cells in soft agar at each step of increasing drug level. This method worked well but did not generate a resistant population any faster than the present method. It should be noted that potent mutagens were not used to generate resistant variants. As the culture adapted to continuous drug exposure, the time between stepwise increase in drug dose decreased, i.e., resistance developed faster. Rapid increase in resistance at the later stages was also reported by Beran & Anderson (1987) during development of resistance to m-AMSA.

Lower drug uptake and/or more efficient efflux often account for the lower drug sensitivity of the resistant cell line (Harker & Sikic, 1985). However, it is difficult to decide what dose of drug to use in uptake experiments since the sensitive and resistant cell lines are so different in their drug sensitivity. The dose should be high enough to cause significant cytoxicity without damaging the efflux mechanism. At 0.3 µg ml$^{-1}$ menogaril, which is the $LD_{90}$ for P388/O and nontoxic to P388/MEN, the difference in uptake and efflux between the 2 cell lines was small. At 1 µg ml$^{-1}$, which is highly toxic ($> LD_{99}$) to P388/O, efflux was very slow, probably because the efflux mechanism was damaged. However, efflux was normal at the 1 µg ml$^{-1}$ dose in P388/MEN, although it was inhibited at 8 µg ml$^{-1}$. Based on the results at 0.3 µg ml$^{-1}$ we concluded that uptake and efflux differences are not likely to explain the difference in sensitivity between P388/O and P388/MEN.

Gene amplification in drug resistant cell lines has been correlated with the appearance of double minutes and HSR in the karyotypes (Robertson *et al.*, 1984). P388/O and P388/MEN had very similar karyotypes with the resistant line having an extra marker chromosome which did have a somewhat variable HSR. It is unlikely that the HSR or small number of double minutes can account for menogaril resistance. This is based on our observation that P388/MEN cells fused with S49 mouse lymphoma cells retain menogaril resistance but do not have the HSR (Badiner *et al.*, 1986*b*).

P388/MEN cells did not meet the established criteria for the multidrug resistant phenotype as seen for other anthracycline resistant cells (Robertson *et al.*, 1984; Kartner *et al.*, 1983). There was not enough difference in uptake or efflux of menogaril to explain the large increase in resistance. Resistance of P388/MEN to menogaril was not reversed by verapmil. P388/MEN cells have a limited pattern of cross-resistance in that they are cross-resistant only to adriamycin but not cross-resistant to cisplatin, *Velban* or actinomycin D. We used the hamster pDR 1.6 DNA probe to show that there was no significant amplification of the *mdr* gene, nor overexpression of this gene as mRNA in P388/MEN. A family of genes has been associated with multidrug resistance and expression of the MDR phenotype. We have investigated one such gene. Other MDR associated genes may, or may not, be important in menogaril resistance in P388 cells.

Several examples of drug resistance to anthracyclines or other natural products (e.g., etoposides, VM-26 or VP-16) that do not involve overexpression of P-glycoprotein have been published. Danks *et al.* (1987) reported the 'atypical' multidrug resistance characteristics of a teniposide (VM-26) resistant human leukaemic (CEM) cell line. They showed that drug resistance was not due to P-glycoprotein-mediated decreased intracellular drug concentration but was probably due to altered interaction between drug and cellular target. Slovak *et al.* (1988) also reported different mechanisms of Adr resistance in 2 human cell lines. Adr resistance in LoVo cells correlated with P-glycoprotein increase whereas a non-P-glycoprotein mediated mechanism operated in HT1080 cells. Resistance to Adr can be multifactorial and result from decreased drug uptake, decreased formation of DNA strand breaks and early onset of repair, increased glutathione transferase activity and elevated P-glycoprotein activity (Deffie *et al.*, 1988). Reduced topoisomerase activity has also been reported to correlate with Adr resistance (Deffie *et al.*, 1989). It is possible that some of these mechanisms may account for menogaril resistance.

Our results clearly show that GSH is not likely to be involved in menogaril resistance in P388/MEN cells. GSH content of P388/MEN cells was not increased over that of the sensitive cell line and the cytotoxicity of menogaril was not potentiated when GSH concentration was lowered by pretreatment with buthionine sulfoximine. Our results suggest that GSH may be involved in adriamycin resistance of P388/ADR cells. P388/ADR cells contained twice as much GSH as the sensitive cell line and lowering GSH content potentiated adriamycin toxicity about 1.5-fold. The GSH content of our P388/O cells was similar to that reported by Ramu *et al.* (1984). They also observed a 1.5-fold increase in GSH and GSH-peroxidase content of P388/ADR cells over that of the sensitive cells. However, they did not see any potentiation of drug cytotoxicity when P388/ADR cells were treated with adriamycin after glutathione depletion by 1-chloro-2,4-dinitrobenzene. Potentiation of adriamycin cytotoxicity by GSH depletion has been reported for human tumour cell lines. Hamilton *et al.* (1985) reported that in both sensitive and adriamycin-resistant ovarian carcinoma cell lines GSH depletion potentiated adriamycin cytotoxicity. Dusre *et al.* (1989) also reported potentiation of adriamycin cytoxicity in multidrug resistant human breast tumour cells. The phenotype of the resistant cell line depends on the cell line and the drug to which it is resistant. The same cell line, P388, made resistant to two different anthracyclines (menogaril or adriamycin) displayed different phenotypes. P388/MEN had a non-multidrug resistant phenotype while P388/ADR fit the established criteria for the MDR phenotype. When the same drug, menogaril, was used to develop two different resistant cell lines, P388/MEN and (Chinese hamster) V79/MEN, the phenotypes were again different. P388/MEN had a non-multidrug resistant phenotype whereas V79/MEN cells had the MDR phenotype (Badiner *et al.*, 1987*a*).

In the light of the difference between P388/MEN and V79/MEN, the clonal heterogeneity of P388/MEN, as expressed by differences in clonogenicity and differences in the level of resistance to menogaril become interesting. The clonal heterogeneity may indicate the existence in P388/MEN of a variety of resistance mechanisms, some of which may be of the MDR type. Such information may be relevant to clinical evaluation of menogaril. The P388/MEN tumour was refractory to therapeutic doses of menogaril and this resistance was stable for at least 12 weeks *in vivo*. Further studies on the *in vivo* cross-resistance pattern of P388/MEN show interesting differences from that seen *in vitro* (Badiner *et al.*, 1987*c*). These observations may be important in the selection of appropriate cell lines for the study of drug resistance.

We thank P. Gros for the pDR 1.6 probe (McGill University, Montreal, Quebec, Canada); R. Ulrich for the electron micrographs, and J. Brewer and W. Adams for HPLC analysis (all of The Upjohn Company, Kalamazoo, MI 49001). We also thank C.A. Kiewiet for secretarial assistance with many copies of this manuscript.

### References

BADINER, G.J. & BHUYAN, B.K. (1986*a*). The evolution and characterization of P388 cells resistant to menogaril. *Proc. Am. Assoc. Cancer Res.*, **27**, 265.

BADINER, G.J., GROPPI, V.E. & BHUYAN, B.K. (1986*b*). Resistance to menogaril is a codominant phenotype in mouse lymphoma cells. *J. Cell. Biol.*, **10**, 28a.

BADINER, G.J., BICHAY, T.J. & BHUYAN, B.K. (1987*a*). Menogaril resistance results in amplification of *mdr* gene in Chinese hamster V79 cells but not in mouse leukemia P388 cells. *Proc. Am. Assoc. Cancer Res.*, **28**, 281.

BADINER, G.J., HAMILTON, R.D., LI, L.H. & BHUYAN, B.K. (1987*b*) Drug sensitivity of ten human tumor cell lines compared to mouse leukemia (L1210) cells. *Inv. New Drugs*, **5**, 219.

BADINER, G.J., WILSON, D.K., LI, L.H. & BHUYAN, B.K. (1987*c*). Comparison of *in vivo* and *in vitro* cross-resistance patterns for P388 sensitive and P388 menogaril resistant cells. *Proc. Am. Assoc. Cancer Res.*, **28**, 299.

BECH-HANSEN, N.T., TILL, J.E. & LING, V. (1975). Pleiotropic phenotype of Colchicine-resistant CHO cells: cross-resistance and collateral sensitivity. *J. Cell. Physiol.*, **88**, 23.

BERAN, M. & ANDERSON, B.S. (1987). Development and characterization of a human myelogenous leukemia cell line resistant to 4'-(9-acridinylamino)-3-methanesulfon-m-anisidide. *Cancer Res.*, **47**, 1897.

BHUYAN, B.K., BLOWERS, C.L. & SHUGARS, K.D. (1980). Lethality of nogalamycin, nogalamycin analogs, and Adriamycin to cells in different cell cycle phases. *Cancer Res.*, **40**, 3437.

CHIRGWIN, J.M., PRZYBYLA, A.A., MACDONALD, R.J. & RUTTER, W.J. (1989). Isolation of biologically active ribonucleic acid from sources enriched in ribonuclease. *Biochem.*, **18**, 5294.

DANKS, M.K., YALOWICH, J.C. & BECK, W.T. (1987). Atypical multiple drug resistance in a human leukemic cell line selected for resistance to teniposide (VM-26). *Cancer Res.*, **47**, 1297.

DEFFIE, A.M., ALAM, T., SENEVIRATNE, C. & 5 others (1988). Multifactorial resistance to Adriamycin: relationship to DNA repair, glutathione transferase activity, drug efflux, and P-glycoprotein in cloned cell lines of Adriamycin-sensitive and -resistant P388 leukemia. *Cancer Res.*, **48**, 3595.

DEFFIE, A.M., BATRA, J.K. & GOLDENBERG, G.J. (1989). Direct correlation between DNA topoisomerase II activity and cytotoxicity in Adriamycin-sensitive and resistant P388 leukemia cell lines. *Cancer Res.*, **49**, 58.

DUSRE, L., MIMNAUGH, E.G., MYERS, C.E. & SINHA, B.K. (1989). Potentiation of doxorubicin cytotoxicity by buthionine sulfoximine in multidrug-resistant human breast tumor cells. *Cancer Res.*, **49**, 511.

FEINBERG, A.P. & VOGELSTEIN, B. (1984). A technique for radiolabelling DNA restriction endonuclease fragments to high specific activity. *Anal. Biochem.*, **137**, 266.

GREEN, J.A., VISTÍCA, D.T., YOUNG, R.C., HAMILTON, T.C., ROGAN, A.M. & OZOLS, R.F. (1984). Potentiation of melphalan cytotoxicity in human ovarian cancer cell lines by glutathione depletion. *Cancer Res.*, **44**, 5427.

GROS, P., GROOP, J., RONINSON, I., VARSHAVSKY, A. & HOUSMAN, D.E. (1986). Isolation and characterization of DNA sequences in multidrug-resistant hamster cells. *Proc. Natl Acad. Sci.*, **83**, 337.

HAMILTON, T.C., WINKER, M.A., LOUIE, K.G. & 7 others (1985). Augmentation of Adriamycin, melphalan, and cisplatin cytotoxicity in drug-resistant and -sensitive human ovarian carcinoma cell lines by buthionine sulfoximine mediated glutathione depletion. *Biochem. Pharmacol.*, **34**, 2583.

HARKER, W.G. & SIKIC, B.I. (1985). Multidrug (pleiotropic) resistance in Doxorubicin-selected variants of the human sarcoma cell line MES-SA. *Cancer Res.*, **45**, 4091.

JOHNSON, R.K., CHITNIS, M.P., EMBREY, W.M. & GREGORY, E.B. (1978). *In vivo* characteristics of resistance and cross-resistance of an Adriamycin-resistant subline of P388 leukemia. *Cancer Treat. Rep.*, **62**, 1535.

KARTNER, N., SHALES, M., RIORDAN, J.R. & LING, V. (1983). Daunorubicin-resistant Chinese hamster ovary cells expressing miltidrug resistance and a cell-surface P-glycoprotein. *Cancer Res.*, **43**, 4413.

KAYE, S. & MERRY, S. (1985). Tumor cell resistance to anthracyclines-a review. *Cancer Chemother. Pharmacol.*, **14**, 96.

KLOHS, W.C., STEINKAMPF, R.W., HAVLICK, M.J. & JACKSON, R.D. (1986). Resistance to anthrapyrazoles and anthracyclines in multidrug-resistant P388 murine leukemia cells: reversal by calcium blockers and calmodulin antagonist. *Cancer Res.*, **46**, 4352.

LI, L.H., KUENTZEL, S.L., MURCH, L.L., PSCHIGODA, L.J. & KRUEGER, W.C. (1979). Comparative biological and biochemical effects of nogalamycin and its analogs on L1210 leukemia. *Cancer Res.*, **39**, 4816.

LING, V. & THOMPSON, L.H. (1974). Reduced permeability in CHO cells as a mechanism of resistance to colchicine. *J. Cell. Physiol.*, **83**, 103.

MANIATIS, T., FRITSCH, E.F. & SAMBROOK, J. (1982). Purification of nucleic acids. In: *Molecular cloning – a laboratory manual*, p. 458. Cold Spring Harbor Laboratory.

McGOVREN, J.P., NEIL, G.L., DENLINGER, R.H., HALL, T.L., CRAMPTON, S.L. & SWENBERG, J.A. (1979). Chronic cardiotoxicity studies in rabbits with 7-con-O-methylnogarol, a new anthracycline antitumor agent. *Cancer Res.*, **39**, 39, 4849.

McGOVREN, J.P. (1980). Activity of the anthracycline agent, 7-con-O-methylnogarol (7-OMEN) administered orally to mice bearing P388 or L1210 leukemia. *Cancer Treat. Rep.*, **64**, 727.

McGOVREN, J.P., HAMILTON, R.D., ADAMS, W.J. & PRATT, E.A. (1984). Quantitation of anthracycline antitumor agent menogarol in plasma using liquid chromatography with fluorescence detection. *Anal. Chem.*, **56**, 1587.

MOY, B.C., TARPLEY, W.G., GROPPI, V.E., BADINER, G.J. & BHUYAN, B.K. (1986). Molecular characterization of P388 mouse leukemia cells resistant to the anthracycline antibiotic, menogaril. *Proc. Am. Assoc. Cancer Res.*, **27**, 300.

NAKASHIMA, K., NISHIDA, K., NAKATSUJI, S. & AKIYAMA, S. (1986). Development and application of organic reagents for analysis. VIII. Determination of biological thiols with a new fluorogenic thiol-selective reagent. N-{p-[2-(6-dimethylamino)-benzofuranyl]phenyl}maleimide. *Chem. Pharm. Bull.*, **34**, 1678.

NEIL, G.L., KUENTZEL, S.L. & McGOVREN, J.P. (1979). Treatment of mouse tumors with 7-con-O-methylnogarol and other analogs of the anthracycline antibiotic, nogalamycin. *Cancer Treat. Rep.*, **63**, 1971.

OTTENBREIT, M.J., HALTON, D.M. & PETERSON, W.D. Jr. (1983). Rapid isoenzyme analysis of cell cultures by agarose electrophoresis. II. Intraspecies identification of human cell lines. *J. Tissue Culture Methods*, **8**, 125.

PETERSON, W.D. Jr., SIMPSON, W.F. & HUKKU, B. (1979). Cell culture characterization, monitoring for cell identification. *Methods in Enzymology*. Vol. **158**, p. 164. Academic Press: New York.

RAMU, A., COHEN, L. & GLAUBIGER, D. (1984). Oxygen radical detoxification enzymes in doxorubicin-sensitive and -resistant P388 murine leukemia cells. *Cancer Res.*, **44**, 1976.

RIORDAN, J.R., DEUCHOVE, K., KARTNER, N., ALON, N., TRENT, J. & LING, V. (1985). Amplification of P-glycoprotein genes in multidrug-resistant mammalian cell lines. *Nature*, **316**, 817.

ROBERTSON, S.M., LING, V. & STANNERS, C.P. (1984). Co-amplification of double minute chromosomes, multiple drug resistance, and cell surface P-glycoprotein in DNA-mediated transformants of mouse cells. *Mol. Cell. Biol.*, Vol. **4**, p. 500.

SCHABEL, F.M. Jr, SKIPPER, H.E., TRADER, M.W., LASTER, W.R. Jr, GRISWOLD, D.P. Jr & CORBETT, T.H. (1983). Establishment of cross-resistance profiles for new agents. *Cancer Treat. Rep.*, **67**, 905.

SLOVAK, M.L., HOELTGE, G.A., DALTON, W.S. & TRENT, J.M. (1988). Pharmacological and biological evidence for differing mechanisms of doxorubicin resistance in two human tumor cell lines. *Cancer Res.*, **48**, 2793.

STERNBERG, C., MAGILL, G., CHENG, E. & HOLLANDER, P. (1986). Phase II trial of menogaril in patients with adenocarcinoma of the pancreas. *Proc. Am. Assoc. Cancer Res.*, **27**, 240.

WEISS, G.R., VON HOFF, D.D., McGOVREN, J.P., NEIL, G.L. & PAQUE, R.E. (1983). Antitumor Activity of 7-Con-O-methylnogarol (7-OMEN) in the human tumor clonogenic assay. *Proc. Am. Assoc. Cancer Res.*, **24**, 309.